# The Impact of Pulmonary Vein Anatomy on the Outcomes of Catheter Ablation for Atrial Fibrillation

**DOI:** 10.3390/medicina55110727

**Published:** 2019-11-04

**Authors:** Sabina Istratoaie, Radu Roșu, Gabriel Cismaru, Ștefan C. Vesa, Mihai Puiu, Dumitru Zdrenghea, Dana Pop, Anca D. Buzoianu

**Affiliations:** 1 Department of Pharmacology, Toxicology and Clinical Pharmacology, “Iuliu Haţieganu” University of Medicine and Pharmacy, 400337 Cluj-Napoca, Romania; sabina.istratoaie@gmail.com (S.I.); stefanvesa@gmail.com (Ș.C.V.); abuzoianu@umfcluj.ro (A.D.B.); 2 5th Department of Internal Medicine, Cardiology-Rehabilitation, “Iuliu Haţieganu” University of Medicine and Pharmacy, 400066 Cluj-Napoca, Romania; rosu.radu1053@gmail.com (R.R.); puiu.mihai@yahoo.com (M.P.); dzdrenghea@yahoo.com (D.Z.); pop67dana@gmail.com (D.P.)

**Keywords:** pulmonary vein anatomy, atrial fibrillation, catheter ablation, outcome

## Abstract

*Background and Objectives:* Prior studies have identified a number of predictors for Atrial fibrillation (AF) ablation success, including comorbidities, the type of AF, and left atrial (LA) size. Ectopic foci in the initiation of paroxysmal AF are frequently found in pulmonary veins. Our aim was to assess how pulmonary vein anatomy influences the recurrence of atrial fibrillation after radiofrequency catheter ablation. *Materials and Methods:* Eighty patients diagnosed with paroxysmal or persistent AF underwent radiofrequency catheter ablation (RFCA) between November 2016 and December 2017. All of these patients underwent computed tomography before AF ablation. PV anatomy was classified according to the presence of common PVs or accessory PVs. Several clinical and imagistic parameters were recorded. After hospital discharge, all patients were scheduled for check-up in an outpatient clinic at 3, 6, 9, and 12 months after RFCA to detect AF recurrence. *Results:* A total of 80 consecutive patients, aged 53.8 ± 9.6 years, 54 (67.5%) men and 26 (32.5%) women were enrolled. The majority of patients had paroxysmal AF 53 (66.3%). Regular PV anatomy (2 left PVs, 2 right PVs) was identified in 59 patients (73.7%), a left common trunk (LCT) was detected in 15 patients (18.7%), an accessory right middle pulmonary vein (RMPV) was found in 5 patients (6.25%) and one patient presented both an LCT and an RMPV. The median follow-up duration was 14 (12; 15) months. Sinus rhythm was maintained in 50 (62.5%) patients. Age, gender, antiarrhythmic drugs, and the presence of cardiac comorbidities were not predictive of AF recurrence. The diagnosis of persistent AF before RFCA was more closely associated with an increase in recurrent AF after RFCA than after paroxysmal AF (*p* = 0.01). Longer procedure times (>265 min) were associated with AF recurrence (*p* = 0.04). Patients with an LA volume index of over 48.5 (mL/m^2^) were more likely to present AF recurrence (*p* = 0.006). Multivariate analysis of recurrence risk showed that only the larger LA volume index and variant PV anatomy were independently associated with AF recurrence. *Conclusion:* The study demonstrated that an increased volume of the left atrium was the most important predictive factor for the risk of AF recurrence after catheter ablation. Variant anatomy of PV was the only other independent predictive factor associated with a higher rate of AF recurrence.

## 1. Introduction

Atrial fibrillation (AF), the most common sustained cardiac arrhythmia, is associated with increased mortality and significant burden of morbidity [1]. Ectopic foci in the initiation of paroxysmal AF are frequently found in pulmonary veins (PV) [2]. These triggers originate from prolongations of the atrial muscle at the PV level with a complex fiber arrangement at the PV junction with the left atrium (LA) [3]. This finding led to an established treatment option for patients with symptomatic AF in the form of radiofrequency catheter ablation (RFCA), which isolates the PV [4]. For persistent AF, besides PV isolation, the atrial substrate modification is also performed.

The success rate after catheter ablation for AF varies as well as the recurrence rate of the atrial arrhythmia after the procedure. Prior studies have identified a number of predictors for AF ablation success, including comorbidities, type of AF, and LA size. Despite this, there is limited data on the potential impact of PV anatomy on ablation outcomes [5]. We hypothesized that given the central role of the PV in triggering and maintaining AF, their anatomical characteristics might have an impact on the RFCA outcome. According to available data, PV anatomy can have different variants, and these include common ostia and additional PVs.

The identification of predictive factors for maintaining sinus rhythm post-ablation would be a significant contribution, as it would help electrophysiologists reduce the number of unnecessary procedures, limit the number of complications, and reduce the costs. The purpose of this study was to determine whether variants of PV anatomy, as determined using CT imaging, can predict the outcome after catheter ablation of AF. 

## 2. Materials and Methods

### 2.1. Patient Selection

The study was a retrospective analytical, observational, longitudinal, and cohort type. Patients with symptomatic, drug-refractory paroxysmal, or persistent AF that underwent the first RFCA of AF in the Cardiology Department of the Rehabilitation Hospital from Cluj Napoca, were enrolled from November 2016 to December 2017. All patients provided written informed consent prior to the procedure. AF was defined as paroxysmal if AF terminated within 7 days of onset (spontaneously or with intervention). If episodes lasted longer than 7 days, AF was defined as persistent type. Exclusion criteria were: reversible causes for AF, the presence of left or right atrial thrombi at transesophageal echocardiography, decompensated heart failure, and a life expectancy of <1 year. The study design was approved by the Ethics Committee of Rehabilitation Hospital Cluj-Napoca (ethical approval no 159/20.4.2016.) and written informed consent was obtained from each patient.

### 2.2. Transthoracic Echocardiogram

All patients underwent transthoracic echocardiographic examinations within 3 weeks from the ablation procedure. The following parameters were recorded: anteroposterior LA diameter, left ventricular end-systolic, and end-diastolic diameters (LVESD and LVEDD respectively), and the left ventricular ejection fraction (LVEF) measured by the Simpson method. All measurements were performed according to the recommendations of the American Society of Echocardiography [6]. All cardiac ultrasound examinations were performed on a Philips Affiniti 50 (Philips Healthcare, Best, The Netherlands) with a 2–4 MHz microconvex transducer.

### 2.3. Analysis of Anatomy

All patients underwent a preablation angiographic CT examination (Optima CT660, GE Healthcare, Milwaukee, WI, USA) within 2 days before the procedure to assess LA and PV anatomy. Angiographic CT images were integrated in the electroanatomical mapping system CARTO 3 (Biosense Webster, Diamond Bar, CA, USA) using the “image integration” software, resulting in 3-dimensional reconstructed images. LA volume was quantitively calculated from the 3-dimenstional reconstructed images and further indexed to the body surface area. All measurements were performed by a blinded investigator with no knowledge of the patient history.

Typical PVs anatomy was defined as two left and two right. Atypical anatomy was determined by the presence of a common trunk or an additional pulmonary vein. The left common trunk (LCT) was defined when the left superior and left inferior PVs joined at least 5 mm before entering the LA, resulting in a single atriopulmonary venous junction [7]. An accessory PV was defined as a supranumerary vein that has its own independent atriopulmonary venous junction separated from the typical superior and inferior PVs and is named for the pulmonary segment that it drains.

### 2.4. The Ablation Procedure

All patients underwent a transesophageal echocardiography within 24 hours prior to RFCA to exclude the presence of LA thrombus. Prior to ablation, anti-arrhythmic drugs (AADs) were discontinued for at least 5 half-lives, while amiodarone was discontinued after more than 2 weeks. To guide all ablation procedures, the three-dimensional NAVX Ensite Velocity (Saint-Jude Medical, Saint Paul, MN, USA) and CARTO 3 (Biosense Webster) mapping systems were used. An open-irrigated 7-french 3.5 mm ablation catheter (Navistar Thermocol, Biosense Webster) or the FlexAbility irrigated ablation catheter (Saint-Jude Medical, Saint Paul, MN, USA) were used to create ablation lesions. The LA access was gained through the transseptal puncture followed by positioning of a circular decapolar mapping catheter within each of the PV antra to assess PV potentials. Continuous radiofrequency ablation was applied at 30–35 W to encircle the ipsilateral PVs until electrical isolation was achieved with a maximum temperature of 45° and an irrigation flow of 20 mL/min. When the circular mapping catheter recorded no PV potential within each antrum, isolation of the PV antrum was considered to be complete. The endpoint of electrical PV isolation was a bidirectional conduction block between LA and PVs. 

In some patients with persistent AF, after PV isolation, additional complex fractioned atrial electrogram (CFAE)-guided or linear ablation were performed based on the operator’s discretion. Further mapping was performed to identify regions of CFAE using an automated mapping algorithm (EnSite Complex Fractionated Electrograms Algorithm—CFE, St. Jude Medical). The algorithm calculated a mean cycle length (CL) of the local electrogram during AF. Regions with a mean CL of less than 120 ms were defined as CFAE and were further targeted for ablation until the local electrogram (EFM) was completely eliminated. CFAE mapping was performed in the LA, coronary sinus (CS) and if AF did not terminate, the right atrium was then mapped and ablated. Other patients with persistent AF underwent additional linear ablation (LA roof line or mitral isthmus) after successful isolation of PV. A minimum of 30 s of RF was delivered at each point to achieve either local EGM elimination or formation of local double potentials. The aim of all linear lesions was to achieve a complete block. The conduction block was confirmed by differential pacing from the CS. 

### 2.5. Follow-Up

During 5 days of hospitalization, sinus rhythm has been closely monitored using daily 12-lead surface ECGs and telemetry. Antiarrhythmic drug therapy (amiodarone, propafenone or flecainide) was chosen by the patient’s physician and it continued for at least 3 months following catheter ablation. After 3 months, it was discontinued or modified at the clinician’s decision according to the evaluation.

After hospital discharge, all patients were scheduled for check-up in an outpatient clinic at 3, 6, 9, and 12 months after RFCA. At every follow-up visit, patients were asked for symptoms of AF and any documented arrhythmia recurrences. Also, ambulatory Holter monitoring was performed for 24 h to detect symptomatic or asymptomatic recurrences at every follow-up visit. They were also encouraged to seek emergency care if they experienced palpitations at any time in order to get the necessary treatment and to obtain ECG documentation. When patients became unable to visit the outpatient clinic of the local center, the follow-up data were obtained by contacting the physicians in-charge of the patients. Any documented atrial arrythmia episode lasting more than 30 s after an initial 3 month blanking period was considered as recurrent AF. 

### 2.6. Statistical Analysis

The MedCalc Statistical Software version 18.11.6 (MedCalc Software bvba, Ostend, Belgium; https://www.medcalc.org; 2019) was used to analyze the data. Quantitative data were checked for normality of distribution (Shapiro-Wilk test, kurtosis and skewness coefficients) and were described using mean ± standard deviation, or median and 25–75%, when appropriate. Qualitative data were expressed as frequency and percentage. Mann–Whitney, Student *t* test or chi-square tests were used for comparisons between groups, when appropriate. ROC curves were used to calculate a cut-off value for quantitative variables, which were associated with the AF recurrence in the univariate analysis. For time dependent AF recurrence, univariate analysis was performed using Kaplan–Meier curves. Multivariate analysis for time dependent AF recurrence was carried out by Cox regression. A *p* value of <0.05 was considered statistically significant. 

## 3. Results

A total of 80 consecutive patients, aged 53.8 ± 9.6 years, 54 (67.5%) men and 26 (32.5%) women were enrolled. The majority of patients had paroxysmal AF 53 (66.3%). The median follow-up duration was 14 (12; 15) months. Out of the 53 patients with paroxysmal AF 73.5% remained in sinus rhythm. The percentage was lower for patients in persistent AF (40.7%). Overall, after the initial blanking period of 3 months, 50 patients (62.5%) had maintained sinus rhythm (nonrecurrence group) and 30 patients (37.5%) had had AF recurrence (recurrence group). No patient has undergone a second ablation for AF recurrence during the follow-up. Regular PV anatomy (2 left PVs, 2 right PVs) was identified in 59 patients (73.7%), a left common trunk (LCT) was detected in 15 patients (18.7%), an accessory right middle pulmonary vein (RMPV) was found in 5 patients (6.25%) and one patient presented both a LCT and a RMPV.

Comparisons between recurrence group and nonrecurrence group can be observed in Table 1.

No differences were found between the typical and variant PV anatomy groups (Table 2).

Patients who had arrhythmia recurrence had a significantly higher prevalence of persistent AF and a lower prevalence of paroxysmal AF. 

The LA volume index was significantly larger in the recurrence group than in the nonrecurrence group. In order to better estimate the risk of AF recurrence, we calculated a cut-off value of 48.5 (mL/m^2^) (AUC 0.713 (CI 95% 0.601–0.809) for the LA volume index, as well as Se 66.7% (CI 95% 47.2–82.7), and Sp 74% (CI 95% 59.7–85.4), *p* < 0.001). 

The procedure time was significantly longer for the patients with AF recurrence when compared to the patients who maintained a sinus rhythm. ROC curve analysis revealed that a cutoff value of 265 min discriminated between patients with and without AF recurrence with a sensitivity of 70% and specificity of 60% (AUC 0657).

With regard to the PV anatomy, there was a significant higher number of patients with variant PV anatomy in the recurrence group than in the non-recurrence group (12 (40%) vs. 9 (18%), *p* = 0.03). No differences in age, sex, comorbidity, or LVEF were observed between those with and without recurrence of AF. 

Before the ablation procedure, patients had been treated with amiodarone (45%), propafenone (40%), and flecainide (15%). No significant association was found between antiarrhythmic medication and the recurrence rate. 

**Figure 1:**
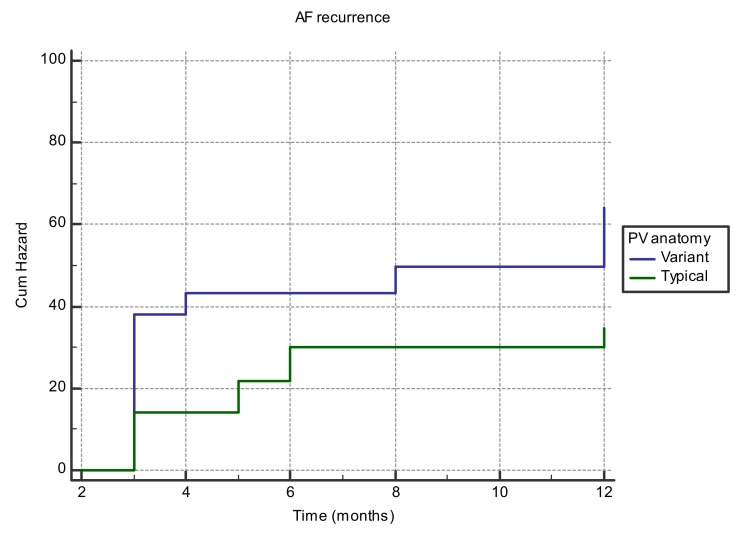
Kaplan Meier curve for AF recurrence risk involving the PV anatomy.

Kaplan-Meier analysis showed that the probability of AF recurrence after initial successful catheter ablation was significantly higher in patients with variant PV anatomy (*p* = 0.01; Figure 1). 

Persistent AF was associated with an increase in recurrent AF after PVI than paroxysmal AF (*p* = 0.01). Longer procedure times (>265 min) were associated with AF recurrence (*p* = 0.04). Patients with LA volume index over 48.5 (mL/m^2^), were more likely to present AF recurrence (*p* < 0.001).

In order to find out which variable was independently associated with the risk of AF recurrence, we used the Cox regression. Multiple models were created, using the data that achieved statistical significance in univariate analysis. Due to the fact that most of the variables were highly correlated to each other, the most stable model included only the anatomy of the PVs and the higher LA volume index (Table 3). Both variables independently predicted the risk of AF recurrence. 

## 4. Discussion

RFCA has become an established treatment option for restoring sinus rhythm in patients with symptomatic AF. Depending on ablation strategy success rates vary between 60% and 80% for paroxysmal AF, while for persistent AF the success rates is between 50% and 60% [8,9]. Our study reported a similar success rate of 73.6% for paroxysmal AF and of only 41% for persistent AF. 

The exact prevalence of atypical PV anatomy is unknown since there are not enough epidemiological data reported. The prevalence of pulmonary vein variants in patients with versus without AF and the studies was compared and the results showed that atypical PV anatomy was much more common in AF patients [10,11,12]. Some studies suggest that PV variant anatomy may play a significant role in the pathophysiology of atrial fibrillation and even increase its incidence [11,12]. The present study showed that 73.8% of the patients had a classical conformation with four pulmonary veins, which was consistent with earlier studies [13,14,15,16]. Of the anatomical PV variants (26.2%), the most common was the presence of a LCT (18.7%), followed by the variant with an accessory right middle pulmonary vein (6.25%). This result is similar to the other studies that also reported that in patients with AF, LCT is the most common variant of PV anatomy [17,18]. Contrary to these outcomes, the study of Mlcochová et al. [19] showed an incidence of typical 4PV in a minority of AF patients (30%). This could be due to a small study population of only 40 patients. On the other hand, the study of McLellan et al. [20] included a larger sample population of 473 patients and reported a typical PV anatomy of 39% in 473 patients, with the atypical PV anatomy being more frequent.

Evaluating the predictive value of PV anatomy on RFCA outcome, the present study showed that patients with atypical PV anatomy had a worse post-ablation evolution with higher AF recurrence risk than those with normal PV anatomy (HR = 2.01). This observation is similar to the study published by Kubala et al. [21] who demonstrated that after 13 months of follow-up, patients with a normal PV pattern had a better outcome in AF recurrence compared to LCT patients (67% vs. 50%). However, the technique used for ablation was not radiofrequency, but cryoballoon ablation. In concordance with our study were the results reported by Sohns et al. [22]. They analyzed the impact of PV anatomy in 146 patients that underwent catheter ablation guided by remote magnetic navigation (RMN) and they showed that the AF recurrence rate is significantly higher in patients with PV variant anatomy vs. typical 4 PV anatomy.

Contrary to the results of the current study, McLellan et al. [20] showed that LCT has a better evolution than the 4 VP group (87% vs. 66%, *p* = 0.03). However, compared to our study, they included only patients with paroxysmal AF. Another recent study of Xu et al. [23] showed that a LCPV was independently associated with better outcomes after repeated RFCA of AF, when compared with typical left-sided PVs. The study also reports that PV reconnection is more frequently identified in cases that present a LCPV anomaly, explaining to some degree the link between this anomaly and the results of repeated ablation.

In the work of Khoueiry et al. a total of 687 patients were enrolled comparing the mid term outcomes of RFCA with those of cryobaloon paroxysmal AF ablation [24]. They did not find a difference in the incidence of AF recurrence (17.0% cryoballoon ablation vs. 14.1% radiofrequency) and no interaction of PV anatomical variants on procedural success was observed. The results of the study suggest that the presence of a left common ostium, as well as an accessory right vein have no significant effect on the outcomes of ablation procedures. They recommend the use of a 28 mm balloon since it allows a wider antral PVI use for both a common ostium and for encompassing accessory veins. Consistent with these findings is a recent study of Mulder et al. [25] that has not found a difference between the typical 4 PV conformation and the other anatomical variants in AF recurrence rate at 12 months follow-up after second-generation cryobaloon ablation use in patients that obtain a AF post-ablation prognosis.

Until recently, there was a hypothesis that fewer pulmonary veins would be easier to isolate, so LCT might lower the AF relapse rate, and an accessory vein would increase the risk of relapse AF post ablation. However, another possible explanation for the results of the studies supporting a more unfavorable evolution for TCS would be that the widened LCT ostium would increase parietal stress around the veno-atrial junction (according to LaPlace’s law), thus leading to differences in wall thickness and to increased resistance to transmural lesions [26]. The many different (sometimes contradictory) outcomes of studies in the effects of PV anatomy on AF clearly show the need for more large-scale research in the subject. 

Our study showed a worse prognosis of AF post-ablation in terms of AF recurrence for a LA volume index >48.5 m vs. <48.5 mm (HR = 3.04). LA volume index was assessed since LA diameter may underestimate LA size. Njoku et al. [27] performed a meta-analysis of nine studies which included over 1425 subjects and demonstrated that the mean LA volume index is higher for patients with AF recurrence after ablation compared to patients with no recurrence. Thirteen other studies of the same meta-analysis demonstrated that LAV/LAVi was an independent predictive factor of AF recurrence postablation with a radiofrequency of (OR-1.032, CI-1.012-1.052).

By comparing the duration of ablation in the two groups with 4 PV or PV anomalies, a significant difference was obtained only in univariate analysis. Hunter et al. [26] analyzed the impact of the PV anatomy for 350 AF patients and found that the duration of ablation increased from 220 min (85–510) in the case of classical conformations of 4 PV to 225 min (110–500) in those with anatomical PV variants (*p* < 0.001). Instead, Sohns et al. [22] followed 138 patients with AF post-ablation and did not obtain a significant difference in treatment duration in patients with 4 PV, 224 ± 49 min, compared to those with PV abnormalities was 255 ± 177 min (*p* = 0.26).

Given the wide variety of catheter ablation results, identifying new predictors of AF recurrence after catheter ablation would allow a better selection of patients to undergo this procedure. Until further studies establish the accuracy of PV anatomy as a predictive factor for AF recurrence, patients with variant anatomy of PV that present AF recurrence should be closely monitored, and advised to undergo a second reablation that may provide significant benefits for rhythm control The current study presented several limitations. Our study included a small group of patients, thus our findings should be validated in a larger population. Using standard out-patient clinic electrocardiogram and 24-hour Holter recordings is likely to underestimate the AF recurrences rate.

## 5. Conclusions

The study demonstrated that an increased volume of the left atrium was the most important predictive factor for the risk of AF recurrence after catheter ablation. Variant anatomy of PV was the only other independent predictive factor associated with a higher rate of AF recurrence. 

## Figures and Tables

**Table 1:** Comparisons between the recurrence group and the non-recurrence group. PV refers to pulmonary veins, AF refers to atrial fibrillation, LA refers to left atrium, CFAE refers to complex fractioned atrial electrogram.

Baseline Characteristics		Recurrence *N* = 30	Non-Recurrence *N* = 50	*p*
Age (years)		52.9 ± 11.2	54.3 ± 8.7	0.5
Sex	M	20 (66.7%)	34 (68%)	1
	F	10 (33.3%)	16 (32%)	
Coronary artery disease		4 (13.3%)	7 (14%)	1
Hypertension		15 (50%)	30 (60%)	0.4
Dyslipidemia		9 (30%)	23 (46%)	0.2
Diabetes Mellitus		2 (6.7%)	4 (8%)	1
Valvular heart disease		20 (66.7%)	29 (58%)	0.4
AF type	Paroxysmal	14 (46.7%)	39 (78%)	0.007
	Persistent	16 (53.3%)	11 (22%)	
Additional intervention for persistent AF	CFAE	3 (18.7%)	1 (9%)	0.6
	Linear ablation	4 (25%)	2 (18.1%)	
	No additional intervention	9 (56.2%)	8 (72.7%)	
LA diameter (mm)		41.8 ± 6	38.4 ± 6.7	0.02
LA volume index (mL/m^2^)		52.7 ± 7.7	47.1 ± 8.9	0.005
LA volume index >48.5 (mL/m^2^)		20 (66.7%)	14 (28%)	0.002
LVESD (mm)		32.2 (26; 37.2)	31.5 (26.5; 37)	0.7
LVEDD (mm)		50 (47.5; 56.2)	49.5 (44.7; 52.5)	0.2
LVEF (%)		55 (50; 60.75)	56.5 (50; 60)	0.8
PV anatomy	Typical PV anatomy	18 (60%)	41 (82%)	0.03
	Variant PV anatomy	12 (40%)	9 (18%)	
Procedure time (minutes)		300 (240; 330)	245 (240; 300)	0.01
Procedure time (minutes)	<265	9 (30%)	29 (58%)	0.02
	>265	21 (70%)	21 (42%)	
Antiarrhythmic drug	Propafenone	11 (36.7%)	21 (42%)	0.7
	Amiodarone	15 (50%)	21 (42%)	
	Flecainide	4 (13.3%)	8 (16%)	

**Table 2:** Comparisons between a typical PV anatomy and a variant PV anatomy group.

Baseline Characteristics		Typical PV Anatomy *N* = 59	Variant PV Anatomy *N* = 21	*p*
Age (years)		54.9 ± 9.5	50.7 ± 9.7	0.08
Sex	M	41 (69.5%)	13 (61.9%)	0.7
	F	18 (30.5%)	18 (30.5%)	
Coronary artery disease		9 (15.3%)	2 (9.5%)	0.7
Hypertension		34 (57.6%)	11 (60%)	0.7
Dyslipidemia		27 (45.8%)	4 (19%)	0.06
Diabetes Mellitus		5 (8.5%)	1 (4.8%)	1
Valvular heart disease		38 (64.4%)	11 (52.4%)	0.4
AF type	Paroxysmal	39 (66.1%)	14 (66.7%)	1
	Persistent	20 (33.9%)	7 (33.3%)	
LA diameter (mm)		39.2 ± 6.5	40.9 ± 6.9	0.3
LA volume index (mL/m^2^)		48.6 ± 8.7	51 ± 9.1	0.2
LVESD (mm)		31 (25; 37)	34 (28; 37)	0.3
LVEDD (mm)		50 (46; 54)	50 (44.7; 55)	0.8
LVEF (%)		60 (50; 60)	55 (50; 59.5)	0.09
Procedure time (minutes)		270 (240; 330)	255 (240; 315)	0.7

**Table 3:** Multivariate analysis for AF recurrence.

Variables	B	P	HR	95.0% CI for HR
Variant anatomy of PV	0.68	0.05	1.9	0.95–4.15
LA volume index >48.5 (mL/m^2^)	1.11	0.004	3.04	1.41–6.55

## References

[1] Krijthe B.P., Kunst A., Benjamin E.J., Lip G.Y., Franco O.H., Hofman A., Witteman J.C., Stricker B.H., Heeringa J. (2013). Projections on the number of individuals with atrial fibrillation in the European Union, from 2000 to 2060. Eur. Heart J..

[2] Haïssaguerre M., Jaïs P., Shah D.C., Takahashi A., Hocini M., Quiniou G., Garrigue S., Le Mouroux A., Le Métayer P., Clémenty J. (1998). Spontaneous initiation of atrial fibrillation by ectopic beats originating in the pulmonary veins. N. Engl. J. Med..

[3] Saito T., Waki K., Becker A.E. (2000). Left atrial myocardial extension onto pulmonary veins in humans: Anatomic observations relevant for atrial arrhythmias. J. Cardiovasc. Electrophysiol..

[4] Oral H., Knight B.P., Tada H., Ozaydin M., Chugh A., Hassan S., Scharf C., Lai S., Greenstein R., Pelosi F. (2002). Pulmonary vein isolation for paroxysmal and persistent atrial fibrillation. Circulation.

[5] Balk E.M., Garlitski A.C., Alsheikh-Ali A.A., Terasawa T., Chung M.P.H. (2010). Predictors of atrial fibrillation recurrence after radiofrequency catheter ablation: A systematic review. J. Cardiovasc. Electrophysiol..

[6] Lang R.M., Badano L.P., Mor-Avi V., Afilalo J., Armstrong A., Ernande L., Flachskampf F.A., Foster E., Goldstein S.A., Kuznetsova T. (2015). Recommendations for Cardiac Chamber Quantification by Echocardiography in Adults: An Update from the American Society of Echocardiography and the European Association of Cardiovascular Imaging. Eur. Heart J. Cardiovasc. Imaging.

[7] Cabrera J.A., Ho S.Y., Climent V., Sánchez-Quintana D. (2008). The architecture of the left lateral atrial wall: A particular anatomic region with implications for ablation of atrial fibrillation. Eur. Heart J..

[8] Ganesan A.N., Shipp N.J., Brooks A.G., Kuklik P., Lau D.H., HS L., Sullivan T., Kurt C., Thomson S., Sanders P. (2013). Long-term outcomes of catheter ablation of atrial fibrillation: A systematic review and meta-analysis. J. Am. Heart Assoc..

[9] Brooks A.G., Stiles M.K., Laborderie J., Lau D.H., Kuklik P., Shipp N.J., Hsu L.-F., Sanders P. (2010). Outcomes of long-standing persistent atrial fibrillation ablation: A systematic review. Heart Rhythm.

[10] Schwartzman D., Bazaz R., Nosbisch J. (2004). Common left pulmonary vein: A consistent source of arrhythmogenic atrial ectopy. J. Cardiovasc. Electrophysiol..

[11] Bittner A., Mönnig G., Vagt A.J., Zellerhoff S., Wasmer K., Köbe J., Pott C., Milberg P., Sauerland C., Wessling J. (2011). Pulmonary vein variants predispose to atrial fibrillation: A case-control study using multislice contrast-enhanced computed tomography. EP Europace.

[12] Skowerski M., Wozniak-Skowerska I., Hoffmann A., Nowak S., Skowerski T., Sosnowski M. (2018). Pulmonary vein anatomy variants as a biomarker of atrial fibrillation–CT angiography evaluation. BMC Cardiovasc. Disord..

[13] Kato R., Lickfett L., Meininger G., Dickfeld T., Wu R., Juang G., Angkeow P., LaCorte J., Bluemke D., Berger R. (2003). Pulmonary vein anatomy in patients undergoing catheter ablation of atrial fibrillation: Lessons learned by use of magnetic resonance imaging. Circulation.

[14] Lacomis J.M., Goitein O., Deible C., Schwartzman D. (2007). CT of the pulmonary veins. J. Thorac. Imaging.

[15] Köse S., Başarıcı I., Kabul K.H., Bozlar U., Amasyalı B. (2012). Catheter ablation of atrial fibrillation in a patient with unusual pulmonary vein anatomy involving right upper pulmonary vein. Anadolu. Kardiyol. Derg..

[16] Stanford W., Breen J.F. (2005). CT evaluation of left atrial pulmonary venous anatomy. Int. J. Cardiovasc. Imaging..

[17] Porres D.V., Morenza O.P., Pallisa E., Roque A., Andreu J., Martínez M. (2013). Learning from the pulmonary veins. Radiographics.

[18] Anselmino M., Blandino A., Beninati S., Rovera C., Boffano C., Belletti M., Domenico C., Scaglione M. (2011). Morphologic analysis of left atrial anatomy by magnetic resonance angiography in patients with atrial fibrillation: A large single center experience. J. Cardiovasc. Electrophysiol..

[19] Mlcochová H., Tintera J., Porod V., Peichl P., Cihák R., Kautzner J. (2005). Magnetic resonance angiography of pulmonary veins: implications for catheter ablation of atrial fibrillation. Pacing Clin. Electrophysiol..

[20] McLellan A.J., Ling L.-H., Ruggiero D., Wong M.C., Walters T.E., Nisbet A., Shetty A.K., Azzopardi S., Taylor A.J., Morton J.B. (2014). Pulmonary vein isolation: The impact of pulmonary venous anatomy on long-term outcome of catheter ablation for paroxysmal atrial fibrillation. Hear. Rhythm..

[21] Kubala M.J.-S., Quenum S., Kubala M., Hermida J., Nadji G., Traulle S., Jarry G. (2011). Normal Pulmonary Veins Anatomy is Associated with Better AF-Free Survival after Cryoablation as Compared to Atypical Anatomy with Common Left Pulmonary Vein. Pacing Clin. Electrophysiol..

[22] Sohns C., Sohns J.M., Bergau L., Sossalla S., Vollmann D., Lüthje L., Staab W., Dorenkamp M., Harrison J.L., O’Neill M.D. (2013). Pulmonary vein anatomy predicts freedom from atrial fibrillation using remote magnetic navigation for circumferential pulmonary vein ablation. Europace.

[23] Xu B., Xing Y., Xu C., Peng F., Sun Y., Wang S., Guo H. (2019). A left common pulmonary vein: Anatomical variant predicting good outcomes of repeat catheter ablation for atrial fibrillation. J. Cardiovasc. Electrophysiol..

[24] Khoueiry Z., Albenque J.-P., Providencia R., Combes S., Combes N., Jourda F., Sousa P.A., Cardin C., Pasquie J.-L., Cung T.T. (2016). Outcomes after cryoablation vs. radiofrequency in patients with paroxysmal atrial fibrillation: Impact of pulmonary veins anatomy. Europace.

[25] Mulder B.A., Al-Jazairi M.I.H., Arends B.K.O., Bax N., Dijkshoorn L.A., Sheikh U., Tan E.S., Wiesfeld A.C.P., Tieleman R.G., Vliegenthart R. (2019). Pulmonary vein anatomy addressed by computed tomography and relation to success of second-generation cryoballoon ablation in paroxysmal atrial fibrillation. Clin. Cardiol..

[26] Hunter R.J., Ginks M., Ang R., Diab I., Goromonzi F.C., Page S., Baker V., Richmond L., Tayebjee M., Sporton S. (2010). Impact of variant pulmonary vein anatomy and image integration on long-term outcome after catheter ablation for atrial fibrillation. Europace.

[27] Njoku A., Kannabhiran M., Arora R., Reddy P., Gopinathannair R., Lakkireddy D., Dominic P. (2018). Left atrial volume predicts atrial fibrillation recurrence after radiofrequency ablation: A meta-analysis. Europace..

